# Sulfonanilide Derivatives in Identifying Novel Aromatase Inhibitors by Applying Docking, Virtual Screening, and MD Simulations Studies

**DOI:** 10.1155/2017/2105610

**Published:** 2017-10-17

**Authors:** Shailima Rampogu, Minky Son, Chanin Park, Hyong-Ha Kim, Jung-Keun Suh, Keun Woo Lee

**Affiliations:** ^1^Division of Applied Life Science (BK21 Plus), Plant Molecular Biology and Biotechnology Research Center (PMBBRC), Systems and Synthetic Agrobiotech Center (SSAC), Research Institute of Natural Science (RINS), Gyeongsang National University (GNU), 501 Jinju-daero, Jinju 52828, Republic of Korea; ^2^Division of Quality of Life, Korea Research Institute of Standards and Science, Daejeon 34113, Republic of Korea; ^3^Bio-Computing Major, Korean German Institute of Technology, Seoul 07582, Republic of Korea

## Abstract

Breast cancer is one of the leading causes of death noticed in women across the world. Of late the most successful treatments rendered are the use of aromatase inhibitors (AIs). In the current study, a two-way approach for the identification of novel leads has been adapted. 81 chemical compounds were assessed to understand their potentiality against aromatase along with the four known drugs. Docking was performed employing the CDOCKER protocol available on the Discovery Studio (DS v4.5). Exemestane has displayed a higher dock score among the known drug candidates and is labeled as reference. Out of 81 ligands 14 have exhibited higher dock scores than the reference. In the second approach, these 14 compounds were utilized for the generation of the pharmacophore. The validated four-featured pharmacophore was then allowed to screen Chembridge database and the potential Hits were obtained after subjecting them to Lipinski's rule of five and the ADMET properties. Subsequently, the acquired 3,050 Hits were escalated to molecular docking utilizing GOLD v5.0. Finally, the obtained Hits were consequently represented to be ideal lead candidates that were escalated to the MD simulations and binding free energy calculations. Additionally, the gene-disease association was performed to delineate the associated disease caused by CYP19A1.

## 1. Introduction

Breast cancer is considered to be one of the leading causes of death in women across the world [1]. More than 2.5 million women in the USA are reported with breast cancer [2]. Majority of the breast cancer cases known today are primarily hormone dependent. The development of aromatase inhibitors has immensely improved the efficacy of the endocrine therapy towards breast cancer. Aromatase enzyme plays a very crucial role in the oestrogen positive breast cancers and hence establishes itself as a promising drug candidate. Delineating the long-term oestrogen deprivation (LTED) narrowed the fact that the breast cancer cells make use of a variety of growth factor pathways and oncogenes to bypass the general endocrine response [3, 4]. The most important signal transduction pathways are the EGFR, HER2, intracellular kinase cascades, transcription genes involved in cell proliferation, and proteins that regulate the cell cycle. Oestrogen receptor positive breast cancer is resistant to tamoxifen [5] and oestrogen receptor positive signaling was assumed to play a paramount role in this. Moreover, the above-mentioned signal pathways may have crosstalk with oestrogen receptor dependent gene transcription [6]. The drugs involved in treating the oestrogen positive breast cancers act either by interfering with oestrogen production or by action. However, aromatase inhibitors act only on the oestrogen produced by breast cancer cells. The rationale behind designing and developing new aromatase inhibitors is to produce a drug molecule with higher clinical efficacy along with reduced side effects that could be beneficial in treating the postmenopausal women diagnosed with breast cancer [7]. However, the success of the use of aromatase inhibitors relies greatly on the mechanism involved in obtaining resistance to aromatase inhibitors and furthermore the cross-talk. Of late, endocrine therapy has failed to some extent in treating the patients with hormone-sensitive breast cancer because the tumors have developed expertise to flee from the endocrine therapy [8], thus developing resistance. Some reasons for obtaining such resistance are due to the upregulation of signal transduction pathways, oestrogen hypersensitivity, and further the cross-talk between the upregulated signal transduction pathways and the oestrogen receptor pathways [6]. In order to overcome this setback duly due to endocrine resistance, one approach is to use the aromatase inhibitors in combination with the signal transduction inhibitors. It was presumed that, by the use of combined therapeutics, it could be made possible for one or several treatments could attack the cancer cells making the treatment more effective [9]. Nevertheless, the prime focus is on identifying the best combination with cost-effectiveness and hence there is a need for developing new aromatase inhibitors. AIs can be grouped into first-, second-, and third-generation drug compounds. The first-generation drugs such as aminoglutethimide have demonstrated a poor selectivity towards CYP450 and were highly toxic. The second-generation drugs with an imidazole group were remarkable and promising as compared with the first-generation drugs; however, they lacked specificity. Later, the third-generation drugs were developed that represented an enhanced therapeutic index and reduced toxicity and therefore were successful in obtaining approval from the FDA [10–12]. The third-generation drugs were further classified into steroidal (type I) and nonsteroidal (type II) inhibitors [13, 14]. The main difference exists with their mechanism of action. Structurally, the steroid inhibitors are the analogues of the substrate androstenedione and thus impart its action by binding irreversibly to the substrate binding site. Such a type of inhibitors is called suicide inhibitors or inactivators [15, 16]. In contrast, the nonsteroidal inhibitors occupy the substrate binding site by interacting noncovalently with the heme [13]. The objective of the present article is to identify most potential aromatase inhibitors from the set of chosen ligands and then utilize them to screen the database to obtain novel lead compounds which could be beneficial in limiting the breast cancer prognosis and further to understand their binding affinities for the establishment of potential drug molecules as compared to the currently used drugs.

## 2. Materials and Methods

**Figure 1:**
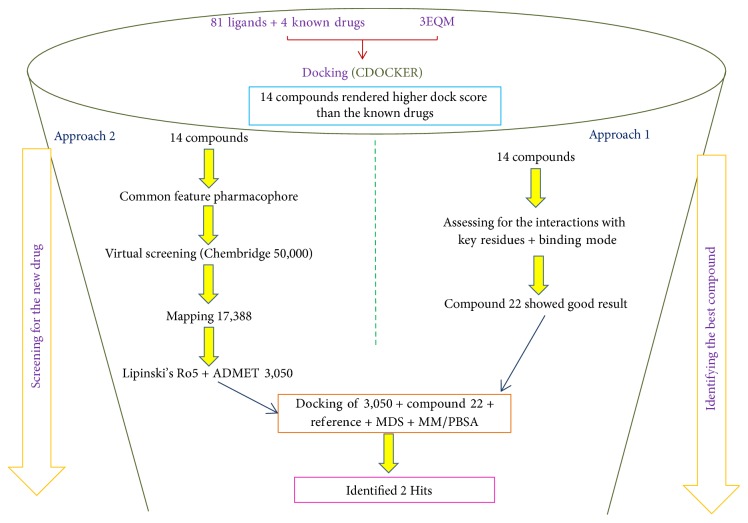
Depiction of the two-step method employed to retrieve the lead candidates from Chembridge database.

To accomplish the objectives, the investigation was executed by a two-step process. The info graphic depiction of the method adapted is shown in Figure 1.

### 2.1. Approach 1

#### 2.1.1. Selection of the Protein

The protein for the present investigation is aromatase (gene: CYP19A1) which was retrieved from the Protein Data Bank (PDB) with the code 3EQM [17, 18] that displayed a resolution of 2.9 Å and is in complex with the substrate 4-androstene-3-17-dione. Protein preparation was performed by removing all the heteroatoms and further the missing hydrogens were added [19]. To make the protein ready for docking, the energy minimization was conducted until satisfied convergence gradient was obtained. The active site was selected around the substrate at 15 Å radius. Additionally, the crucial residues located at the active sites were identified as Arg115, Ala306, Asp309, Val370, Leu372, Met374, and Leu477 [20]. Among them, the amino acid residue Met374 has been identified to play an important role by forming the hydrogen bond interactions with the substrate molecule. The other residues impart electrostatic and hydrophobic interactions. Therefore, the interactions with these residues are imperative in identifying novel lead molecules.

#### 2.1.2. Selection of the Ligands and Preparation

**Figure 2:**
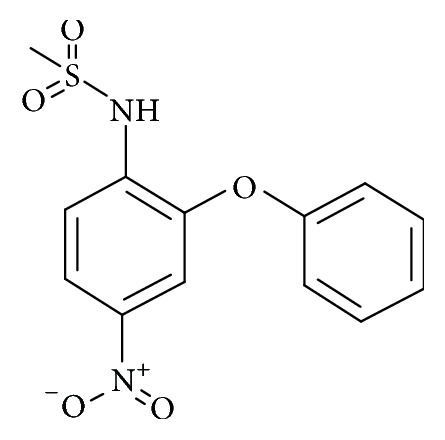
General structure of nimesulide.

**Figure 3:**
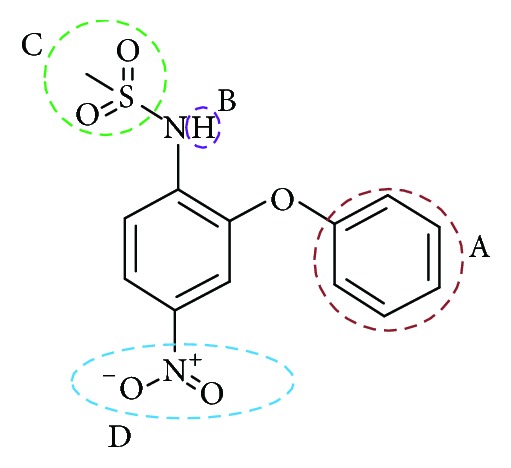
Nimesulide with modifications.

Sulfonanilide derivatives are known to suppress aromatase activity in transfected MCF-7 cells [1]; however their binding potency has not been tested. Therefore several sulfonanilide derivatives were used for the present investigation. 81 sulfonanilide derivatives were drawn on the Marvin sketch tool (https://www.chemaxon.com/products/marvin/marvinsketch/), Supplementary 1, in Supplementary Material available online at https://doi.org/10.1155/2017/2105610. Further, energy minimization with CHARMm force field was applied. The selected 81 compounds were designed based on nimesulide as described by Su et al., Figures 2 and 3 [1], and were imported onto the DS v4.5. Additionally, the known inhibitors, namely, anastrozole, exemestane, letrozole, and tamoxifen, were also sketched on Marvin sketch and were subsequently imported onto the DS v4.5.

### 2.2. Approach 2

The prospective drug compounds that have displayed the higher dock scores than the known inhibitors (higher one among them is labeled as the reference molecule) were employed to generate the pharmacophore model employing the* HipHop common feature pharmacophore generation* protocol implemented on the DS. HipHop specifically extracts the common feature pharmacophore models utilizing the information available on the set of given active compounds. The interfeature distance was taken as 2.97, while keeping all the other features as default.

#### 2.2.1. Validation of the Generated Pharmacophore

The generated pharmacophore was further validated to assess its predictive ability in retrieving the active molecules from the database used for screening. Accordingly, the generated pharmacophore was validated using the ROC curve and the decoy set method. The ROC curve was performed alongside the pharmacophore generation. In the ROC method a set of ligand molecules were taken consisting of known active and known inactive compounds. A total of 20 molecules were selected that consisted of 14 known active compounds and the remaining were the known inactive compounds. In the decoy set validation [21], a dataset of 107 molecules was instituted comprising the 14 active compounds. The* ligand pharmacophore mapping* protocol available on the DS was employed to understand the ability of the pharmacophore model to map with the active compounds. Subsequently, the GH and the EF [21] values were computed to affirm the same.

#### 2.2.2. Database Screening and Drug-Like Properties

Pharmacophore based virtual database screening is one of the most advanced methods used to identify the novel potential leads for further development. A pharmacophore thus imbibes all the bioactive features of the prospective drugs and is therefore recommended to screen the databases to obtain the candidate compounds. The validated pharmacophore was therefore adapted to screen the Chembridge database in order to obtain the lead molecules that oblige to all the pharmacophoric features. The database screening was initiated employing the* ligand pharmacophore mapping* protocol available on the DS using the* best flexible* algorithm. The retrieved Hit molecules that mapped with all the features were subjected to Lipinski's rule of five and the ADMET implemented on the DS.

#### 2.2.3. Molecular Docking

CDOCKER, available with the DS v4.5, was recruited for analyzing the binding affinities between the protein and the ligand that relies largely on the CHARMm force field. Consequently, diverse conformations were generated adapting the random rigid body rotation. Each ligand was allowed to generate 30 conformers, keeping all the other parameters as default. The docking results were read by the -CDOCKER interaction energy [22, 23], which implies the energy of the nonbonded interactions that exists between the protein and the ligand [19, 24]. Additionally, higher -CDOCKER interaction energy values denote greater favorable binding between the protein and the ligand [19, 24]. Docking was performed with 81 ligand molecules along with four FDA approved breast cancer drugs, to understand the potency of the new drugs. The known drugs used for the present investigation are anastrozole, exemestane, letrozole, and tamoxifen. The prepared protein and the ligands were imported onto DS v4.5 for the execution of the docking protocol. Corresponding results were evaluated based upon the -CDOCKER interaction energy, hydrogen bond interaction, and the binding mode pattern.

#### 2.2.4. Molecular Dynamics Simulations

To further affirm the potentiality of the selected compounds and to evaluate the dynamic behaviour of the prospective drug molecules in the binding site pocket, they were subjected to MD simulation along with the reference compound using GROMACS 4.5.7 [25–27], employing CHARMm27 [25] force field. Ligand topologies were generated using SwissParam [28]. Corresponding counterions were added to neutralize the solvated TIP3P water model present in the dodecahedron box. Unwanted contacts from the initial structure were dislodged by performing the energy minimization, adapting the steepest descent algorithm which was followed by the NVT and NPT equilibration steps. During this process, the solvent molecules along with the counterions were allowed to move restraining the protein backbone. Both the processes were executed by 100 ps at 300 K and a pressure of 1 bar, respectively. Parrinello-Rahman barostat was employed to maintain the pressure of the system [29]. The geometry of the water molecules and the bonds involving hydrogen atoms were constrained employing SETTLE and LINCS, respectively [30, 31]. Particle Mesh Ewald (PME) method [32] was used to calculate long-range electrostatic interactions. A cut-off distance of 12 Å was attributed for Coulombic and van der Waals interactions. The equilibrated structures were then subjected to production MD conducted for 50 ns and the results were evaluated on VMD [33] and DS.

#### 2.2.5. Time Based Binding Free Energy Calculations

To delineate further the protein-ligand complex, time based binding free energy calculations were performed. Molecular Mechanics/Poisson-Boltzmann Surface Area (MM/PBSA) [34, 35] was used for its accomplishment and was performed after the MD simulations. The obtained Δ*G* takes into account the protein fluctuations and the ligand conformations, which therefore ensures proper positioning of the ligand within the binding pocket.

The binding free energy protein-ligand complex in solvent system is stated as

(1) $$\begin{array}{c} \Delta G_{binding}=G_{complex}-\left(\;G_{protein}+G_{ligand}\;\right). \end{array}$$

Herein, *G*_complex_ refers to the total free energy of the complex and *G*_protein_ and *G*_ligand_ indicate the separated protein and ligand in the solvent. Their free energies can be computed by

(2) $$\begin{array}{c} G_{X}=E_{MM}+G_{solvation}, \end{array}$$

where *X* can be a protein, ligand, or its complex. *E*_MM_ represents the average molecular mechanics potential energy in vacuum, while *G*_solvation_ interprets the free energy of solvation.

Additionally, molecular mechanics potential energy in vacuum can be evaluated by adapting the equation

(3) EMM=Ebonded+Enon-bonded=Ebonded+Evdw+Eelec.

*E*_bonded_ represents the bonded interactions, while the nonbonded interactions are denoted by *E*_non-bonded_. Δ*E*_bonded_ is generally regarded as zero [36].

The solvation free energy (*G*_solvation_) is expressed by the sum of electrostatic solvation free energy (*G*_polar_) and apolar solvation free energy (*G*_non-polar_) and is given as follows:

(4) $$\begin{array}{c} G_{\text{solvation}}=G_{polar}+G_{\text{non-polar}}. \end{array}$$

*G*_polar_ is computed recruiting the Poisson-Boltzmann (PB) equation [37] while *G*_non-polar_ is computed from the solvent-accessible surface area (SASA) and can be written as follows:

(5) $$\begin{array}{c} G_{non\_polar}=\gamma SASA+b. \end{array}$$

Here, *γ* is the coefficient of the surface tension of the solvent whereas, *b* is its fitting parameter, whose values are 0.02267 kJ/mol/Å^2^ or 0.0054 kcal/mol/Å^2^ and 3.849 kJ/mol or 0.916 kcal/mol, respectively.

#### 2.2.6. Gene Network and Disease Complexity

To further understand the complexity of the disease in terms of knowing the association of the causative gene with other genes and diseases, a systematic gene-disease association and gene-gene association was performed employing the DisGeNET [38, 39]. DisGeNET efficiently finds the related genes to the disease and vice versa. This investigation was undertaken to delineate the diseases caused by the same gene such that there might be a possibility of using the same drugs that were identified. Several reports exist on such strategies and have gained increasing popularity [40–43]. The development of such methods has certain advantages such as being time- and cost-effective [44, 45].

For the current investigation, the gene CYP19A1 has been used as a query to examine the diseases and genes associated with it. The results are read as scores to rank based on the supporting evidence. Score is the accuracy of the gene-disease pair depending upon the type and the number of sources reported from PubMed.

## 3. Results

### 3.1. Approach 1

#### 3.1.1. Molecular Docking Mechanism

**Figure 4:**
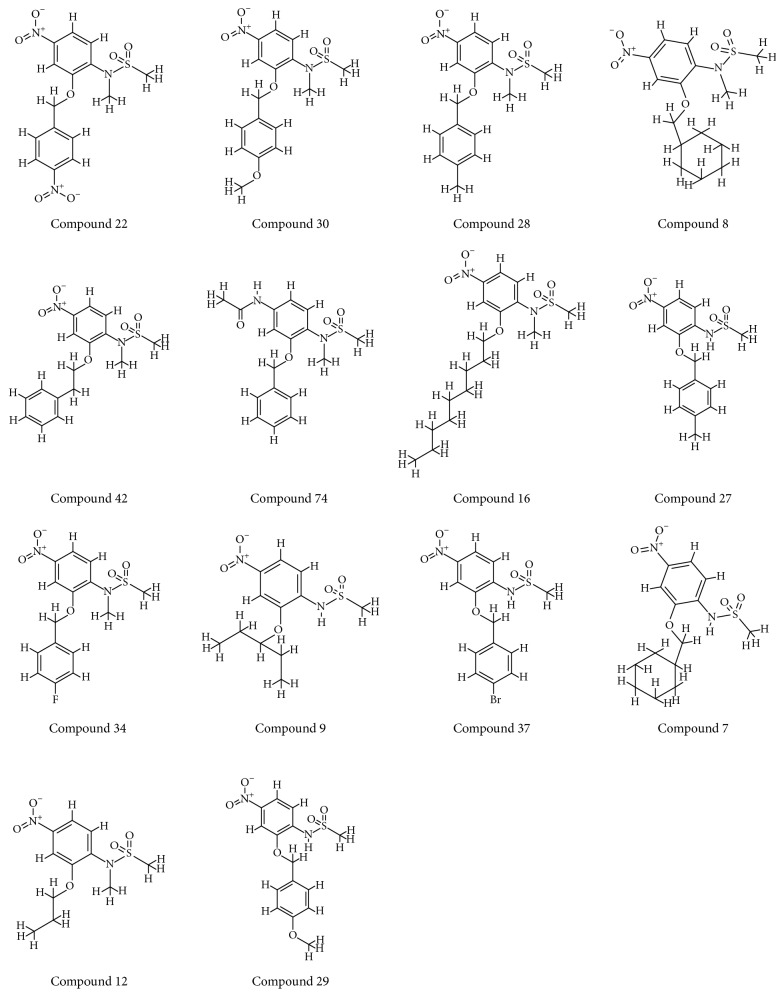
2D structures of the inhibitors that showed higher dock score than the reference compound. The figures are placed in the order of their CDOCKER interaction energies.

**Figure 5:**
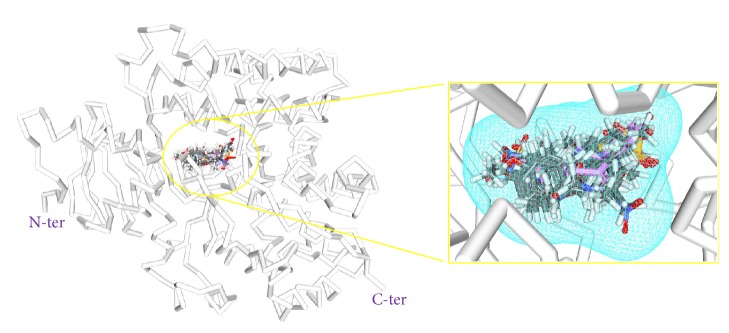
Binding patterns of 14 inhibitors along with the reference compound (pink) and the cocrystal (blue). Blue mesh indicates the closed surface. Picture on the left demonstrates the overlying of the compounds and picture on the right is its enlarged form.

The selected ligands along with the known inhibitors have rendered good docking results when challenged against the protein drug target. The docking results showed that the CDOCKER interaction energies of the 81 compounds were higher than the known drug candidates as represented in Table 1 and Supplementary 2. The -CDOCKER interaction energies of the known drugs have ranged between 29 kcal/mol and 49 kcal/mol. Among them, exemestane has displayed a higher score of 48.27 kcal/mol. Therefore, the -CDOCKER interaction energy of this known drug was considered as reference to screen 81 ligands, Table 1 and Supplementary 2. Subsequently, further studies were performed taking exemestane as a reference molecule. This is one indication that proves the efficacy of the 81 ligands. Accordingly, 14 inhibitors have shown higher dock score than the exemestane, Table 1 and Figure 4. Furthermore, it was observed that the 14 inhibitors have aligned in the same binding pattern as that of the cocrystal and the reference compound, Figure 5 and Supplementary 3. The obtained 14 compounds were used to generate the pharmacophore model for the subsequent extraction of the novel inhibitors against the aromatase enzyme, a process that occurs in approach 2.

### 3.2. Approach 2

#### 3.2.1. Pharmacophore Generation

**Figure 6:**
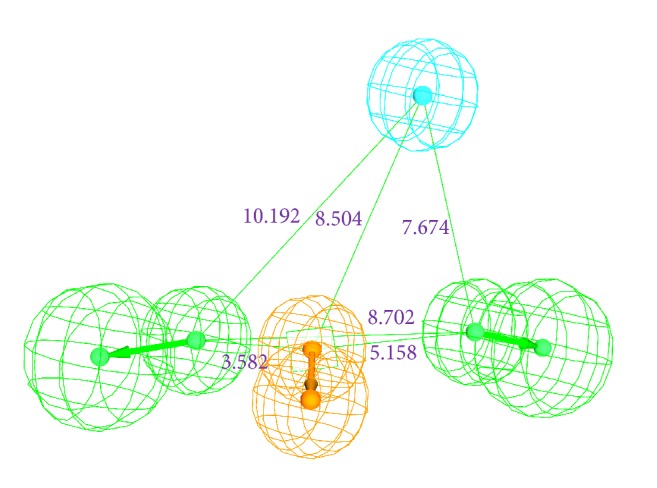
Pharmacophore model with four features and its geometry. Green represents the hydrogen bond acceptor; orange denotes the aromatic ring and cyan refers to hydrophobic feature.

**Figure 7:**
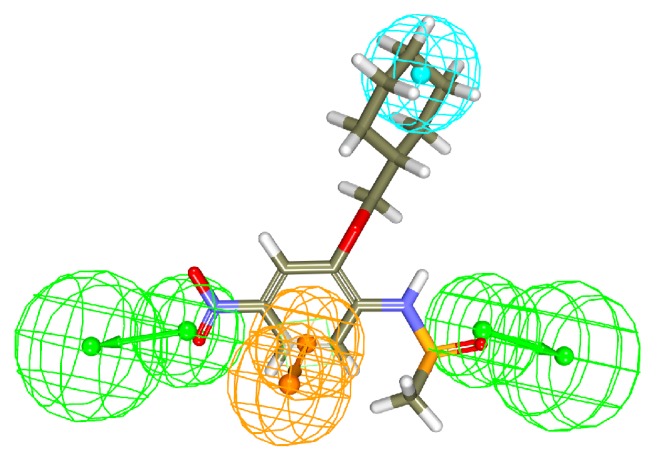
Overlay of the most active compound onto the four-featured pharmacophore. The compound was shown to map with all the four features.

The 14 compounds that have generated the higher dock scores were employed to generate the pharmacophore model using the* common feature pharmacophore* protocol implemented on the DS. These 14 compounds exhibited a range of IC_50_ values and were structurally diverse. All the parameters were selected as default and selection of the best pharmacophore hypothesis was based upon the rank and the maximum number of features showed by the hypothesis. Out of the 10 generated pharmacophores, seven have displayed one ring aromatic, two hydrogen bond acceptors, and one hydrophobic feature, while the remaining three have displayed one hydrophobic and three hydrogen bond acceptors, Supplementary 4. Furthermore, it can therefore be understood that the hydrophobic and the hydrogen bond acceptors are the most important features. Subsequently, a pharmacophore comprising four features was generated consisting of one hydrophobic, one aromatic ring, and two hydrogen bond acceptors; Figure 6 was selected as it displayed a higher rank of 134.98. The ranking is a measure of how well the molecules map onto the proposed pharmacophores, as well as the rarity of the pharmacophore model. The best pharmacophore model is more likely to map with the active compounds thus generating the higher rank. Further screening of the database was performed based on the fit value ≥ 4. Additionally, the most active compound from the test set was superimposed onto the pharmacophore. It was noticed that the most active compound (compound 7) has mapped with all the features of the pharmacophore, Figure 7.

#### 3.2.2. Validation of the Pharmacophore

**Figure 8:**
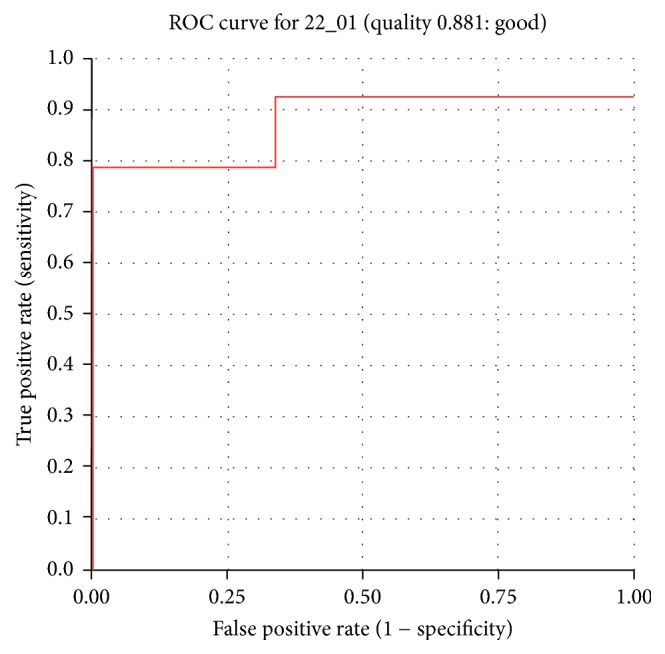
ROC curve obtained considering 14 higher docked score compounds as known active compounds.

The generated pharmacophore was validated by utilizing the ROC curve, which was computed simultaneously during the pharmacophore generations and by the decoy set method. The ROC curve validation was proceeded by taking the known 14 active compounds and the 6 known inactive compounds. The 6 known inactive compounds were systematically chosen and are shown in Supplementary 5. Logically, the chosen pharmacophore should map with the active compounds leaving away the known inactive compounds. The generated plots imply the accuracy of the model in distinguishing between true positives and the true negatives. The resultant ROC plot indicated that the pharmacophore model was efficient in identifying the true positives from the true negatives and thus the quality of the model was pronounced to be good with 0.88, Figure 8. As a second method, the decoy set method was initiated, Table 2, for which an external dataset (*D*) comprising 107 compounds was instituted, consisting of 14 active compounds. The ligand pharmacophore mapping protocol available on the DS was executed. The Güner-Henry (GH) scoring method was used to evaluate the pharmacophore hypotheses and was calculated employing the formula GH = {[Ha *∗* (3*A* + Ht)]/(4Ht*A*)}*∗*[1 − (Ht − Ha)/(*D* − *A*)]. The GH score may range from 0 to 1 signifying the quality of the model to be between null and ideal [46]. Consequently, the pharmacophore could map with a total of 15 Hits (Ht) which had 13 active molecules (Ha) and the corresponding GH score was calculated to be 0.76 indicating the generated pharmacophore model to be a good one [47]. The other results of the decoy set validation are tabulated in Table 2. The quality of the pharmacophore model was proved by the two validations and further this affirms that the model has an ability to retrieve the active molecules from a given database.

#### 3.2.3. Virtual Screening of the Chembridge Database

The validated pharmacophore has been used as a query to screen the Chembridge database in pursuit of obtaining the lead molecules.* Ligand pharmacophore mapping* protocol was initiated to screen the database containing 50,000 compounds during which all the parameters were set as default. The pharmacophore model was successful in mapping with 17,388 compounds, which were forwarded to Lipinski's Ro5 and the ADMET studies. Rule of five (Ro5), according to Christopher A. Lipinski, is a rule of thumb to determine if a biologically active compound possesses the properties that could make it an ideal orally active drug in humans [48]. Therefore, an ideal chemical compound should have no more than 5 hydrogen bond donors, less than 10 hydrogen bond acceptors, log⁡*P* no greater than 5, and a molecular weight less than 500 Daltons. Additionally, the ADMET properties were calculated to understand the pharmacokinetics of a prospective drug molecule in the human body. Accordingly, the blood brain barrier (BBB) penetration, hepatotoxicity, solubility, adsorption, plasma protein binding (PPB), and CYP450 2D6 inhibition were assessed. Corresponding upper limit for the BBB, solubility, and the absorption were duly set as 3, 3, and 0. A total of 3,050 compounds have obeyed the aforementioned criteria and were escalated to molecular docking studies to further gain insight into their binding modes.

#### 3.2.4. Molecular Docking Mechanism

**Figure 9:**
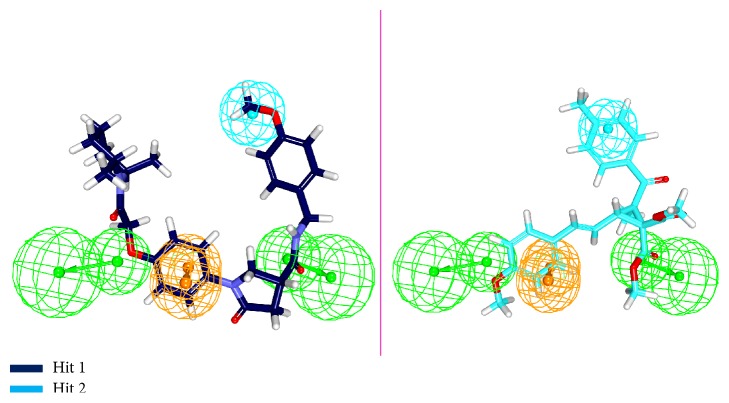
Overlay of the Hits onto the pharmacophore features. The Hits are seen to map with all the features of the pharmacophore.

Molecular docking protocol was initiated as described earlier with the screened 3,050 compounds along with compound 22 (obtained from approach 1) and the reference compound. All those compounds that have displayed a higher CDOCKER interaction energy than the reference and the ligands that satisfied the necessary interactions with the active site compounds were chosen, Table 3. Eventually two compounds have obeyed the above criteria and have mapped well with the pharmacophore features, Figure 9. These Hits were therefore forwarded to the MD simulations and further the binding free energy calculations.

#### 3.2.5. Molecular Dynamics Simulations

**Figure 10:**
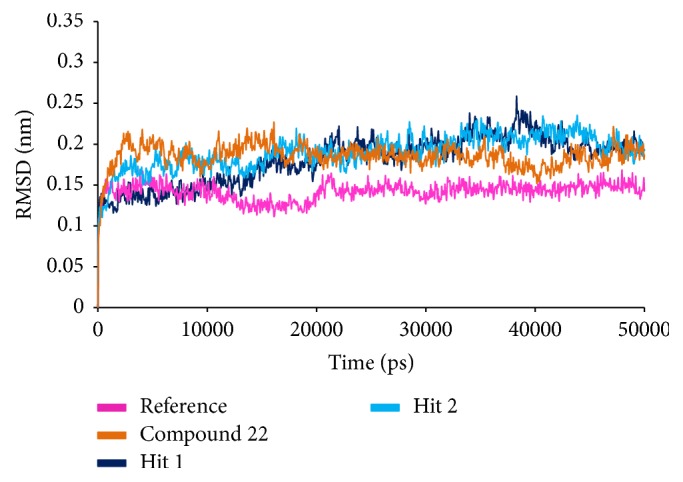
RMSD plot to assess the overall stability of the system for 50 ns of MD simulations.

**Figure 11:**
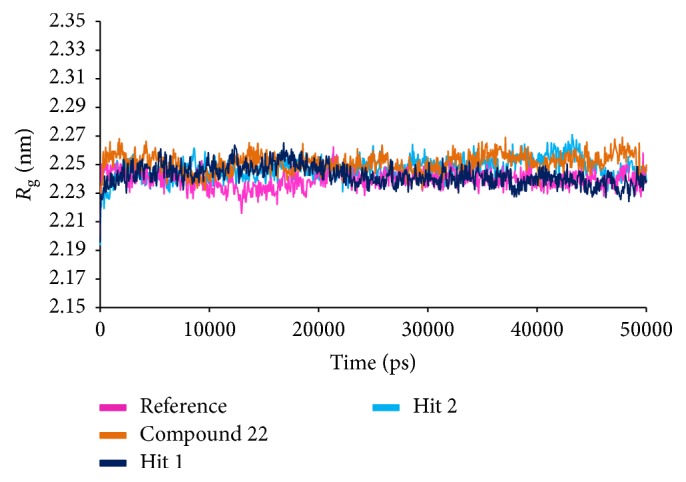
Radius of gyration profiles of the reference and the Hits.

**Figure 12:**
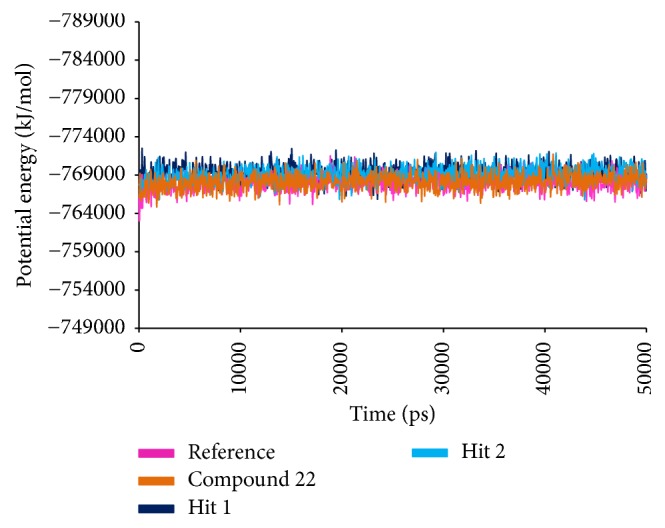
Potential energy of the reference and the Hits. All the systems are well converged.

**Figure 13:**
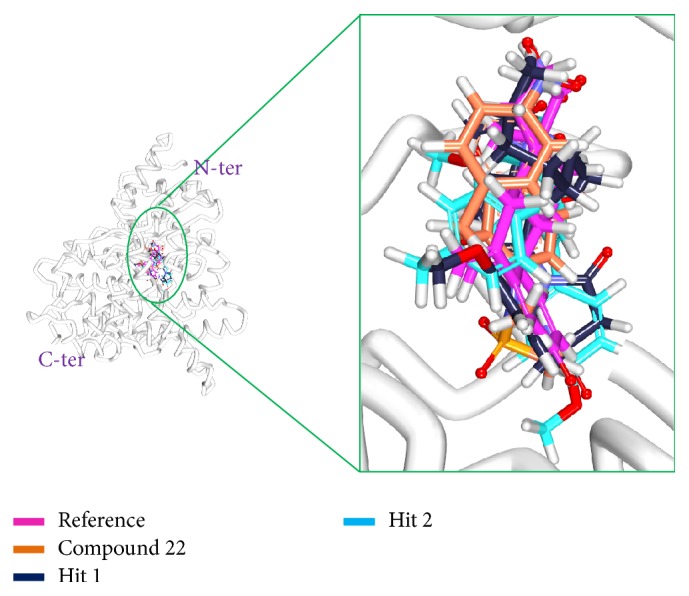
Binding mode analysis of the reference and the Hits. Picture on the left represents the superimposed structure and on the right is its enlarged form.

**Figure 14:**
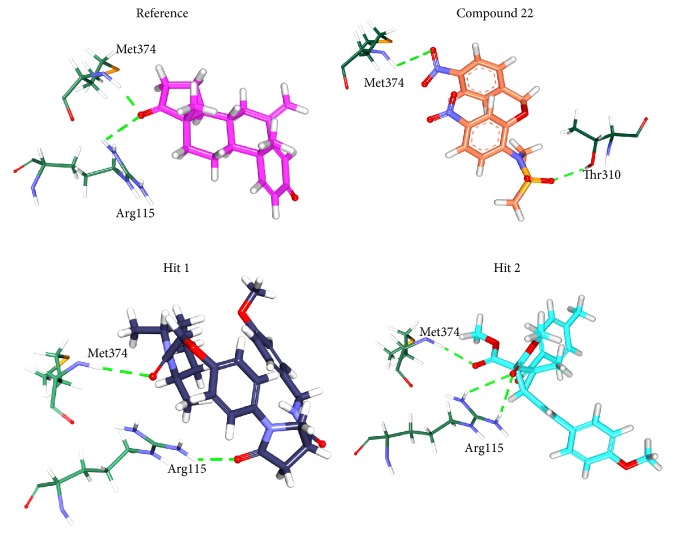
Hydrogen bond interactions between the protein and the ligands. Green dashed lines indicate the hydrogen bonds.

**Figure 15:**
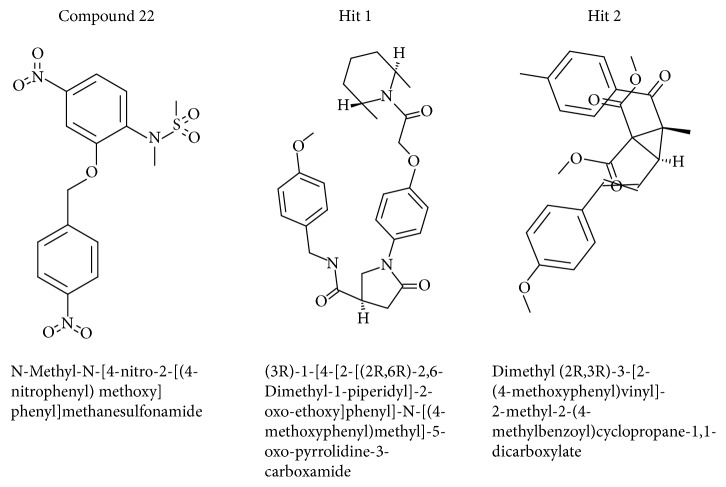
Two-dimensional structures of the Hit compounds. The IUPAC name is provided for each compound.

To further validate the superiority of compound 22 and the Hits, they were forwarded to the MD simulation and binding free energy calculations along with the reference compound. MD run of 50 ns was therefore performed to elucidate the conformational variation, their behaviour, and further their binding stability. Accordingly, the RMSD calculations were executed for the reference and Hits for the protein backbone atoms to assess their overall stability. The RMSD of the reference, compound 22, Hit1, and Hit2 were found to be 0.16 Å, 0.2 Å, 0.27 Å, and 0.2 Å, respectively, during 50 ns MD run. Additionally, the RMSD further revealed that the stability for all the four systems was attained after 12,000 ps without any further deviation thereafter, Figure 10. The stability of the protein was additionally assessed by computing the potential energy and the radius of gyration. The radius of gyration enables understanding the compactness of the protein [49, 50] and thus its folding. The result, Figure 11, is suggestive that compound 22 and the Hits were stably folded throughout the MD simulation and was similar to the reference. Moreover, the potential energy of Hits and the reference compound were seen to be unvarying throughout the simulation, Figure 12. The binding mode analysis was conducted on last 5 ns trajectories for the reference, compound 22, and the Hits, correspondingly. Upon superimposition of the corresponding representative structures of the reference and the Hits, it was noticed that the reference and the Hits converged in similar pattern, Figure 13. Moreover, the intermolecular interactions of the reference and the Hits have revealed the presence of the key residues, Table 4 and Figure 14. The reference molecule has formed two hydrogen bonds with Arg115 and Met374 correspondingly. The HH11 of Arg115 has joined with the O1 of the reference compound with a bond length of 2.7 Å and the HN of Met374 has interacted with O1 of the exemestane with an acceptable bond length of 1.8 Å. Further delineating the intermolecular interactions reveals that the Phe134, Ile305, Ala306, Asp309, Thr310, Leu372, Leu477, and Ser478 have been involved in the van der Waals interaction and Ile133, Trp224, Trp224, Val370, and Val373 have formed the alkyl/*π*-alkyl bonds. Compound 22 has rendered hydrogen bond interactions with two key residues, Thr310 and Met374, and was represented by two hydrogen bonds. HN atom of Met374 has formed hydrogen bonds with the ligand O26 atom displaying a bond length of 2.8 Å, respectively. HG1 atom of Thr310 has interacted with O13 atom of the ligand with a bond distance of 2.2 Å. Moreover, the Phe134, Phe221, Trp224, Asp309, Val374, Leu372, and Ser478 have interacted with the van der Waals interactions. The alkyl/*π*-alkyl bonds were formed with Ile133 and Val370, respectively. Hit1 has formed two hydrogen bond interactions with Met374 and Arg115 with a bond length of 2.7 Å and 2.2 Å, respectively. The NH atom of the Met374 was observed to join with the O16 of the ligand and the HH21 atom of the protein has interacted with the O11 of the ligand. The HH21 atom of Arg115 has interacted with O11 atom of the ligand with a bond length of 2.2 Å. Furthermore, Hit2 has formed three hydrogen bonds with Met374 and Agr115. Met374 has exhibited one hydrogen bond with HN atom and O11 of the ligand with a bond distance of 2.2 Å. The O12 atom of Hit2 has formed hydrogen bonds with HH12 and HH22 atoms of Arg115 with a bond distance of 2.8 Å and 2.4 Å, respectively. The details of the interactions are tabulated in Table 4 and the corresponding 2D structures of the Hits are represented in Figure 15. Furthermore, their systemic IUPAC names were generated from Biovia draw and are represented in Figure 15.

#### 3.2.6. Time Dependent Binding Free Energy Calculations

**Figure 16:**
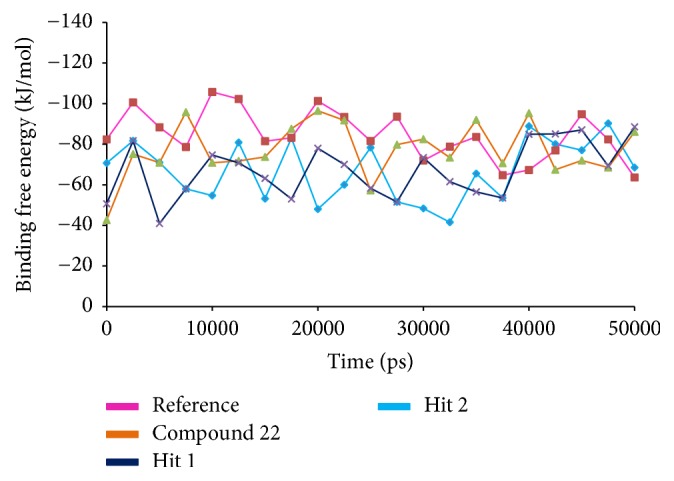
MM/PBSA estimated binding free energies of the corresponding systems.

**Figure 17:**
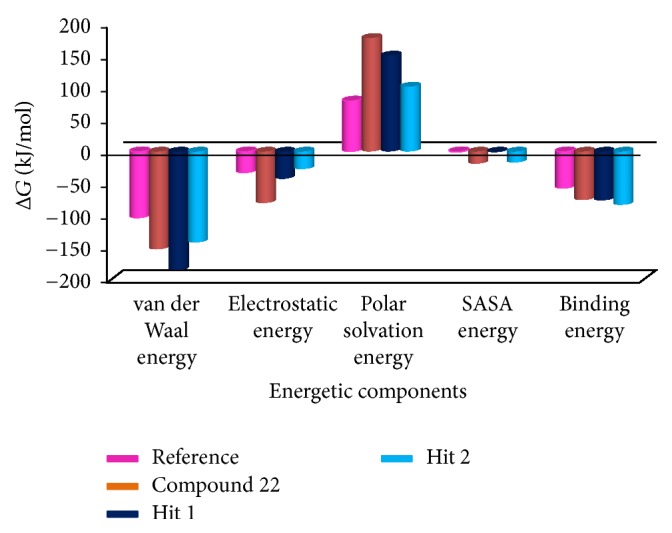
Graphical demonstration of different energetic components computed by MM/PBSA.

MM/PBSA was adapted for the execution of the binding free energy calculations. 20 snapshots were evenly generated from 1 to 50 ns MD trajectories [51, 52]. The binding free energy value, Δ*G*, is the sum of protein fluctuations in the complex and the conformations of the ligand, a measure employed to secure an appropriate positioning of the ligand within the binding site [53, 54]. To accomplish this step, the MM/PBSA was executed which renders information on van der Waals energy, electrostatic energy, polar solvation energy, SASA energy, and the binding energy. The corresponding results generated favorable Δ*G* values for the reference and the Hits that ranged between −80 kJ/mol and −40 kJ/mol as depicted in Figure 16. Minor deviations in the corresponding snap shots were observed as the conformational space was not sampled enough to render the converged results [55]. These findings showed that the Hits have displayed a favorable van der Waals energy, electrostatic and polar solvation, and SASA energetic terms as compared with the reference compound. Moreover, the average binding energy of the reference was computed to be −56.88 kJ/mol, while compound 22, Hit1, and Hit2 have exhibited −77.20 kJ/mol, −77.77 kJ/mol, and −82.58 kJ/mol, respectively. The polar solvation was found to have been contributed positively to binding energies, Figure 17.

#### 3.2.7. Gene Network and Disease Complexity

To further understand the association of aromatase gene with different disease, the query was given as CYP19A1. Information pertaining to top 10 diseases associated with the query gene and the top 10 genes that share diseases with this gene was retrieved, Tables 5 and 6. CYP19 has been involved with multiple diseases related to women and more particularly was seen to be associated with cancer. However, osteoporosis was also found to be influenced by CYP19 that is predominant in elderly women, Table 5. The same has been noted when probing into the genes that share diseases with CYP19. Additionally, it was evident that the CYP19 is crucial in several cancer aliments, Table 6.

## 4. Discussion

**Figure 18:**
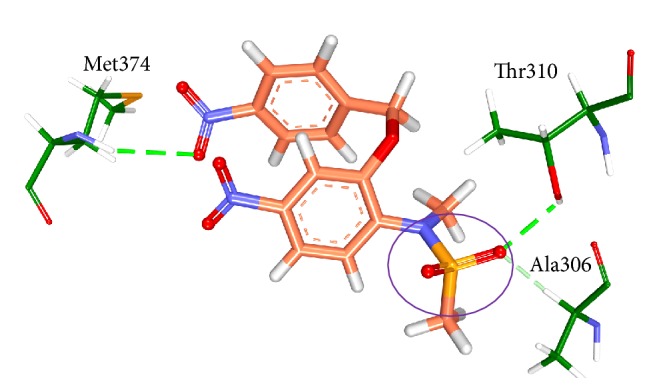
Pictorial elucidation of the sulfonamide moiety of compound 22. Thr310 and Ala306 residues are involved with the O13 atom of the sulfonamide inhibitor group.

With an objective of identifying most potential aromatase inhibitors, the article proceeds taking into consideration the small molecules as described by Su et al. and accordingly they were drawn on Marvin sketch. CDOCKER protocol available on the DS v4.5 was recruited to perform the docking along with the known breast cancer inhibitors. The produced dock results were analyzed based on the -CDOCKER interaction energy; 14 ligands have displayed the higher dock scores as compared to the known drugs. Therefore, these 14 drug candidates have been employed to generate the pharmacophore model of superior quality. Additionally, the best candidate from the 14 compounds was considered for further studies. Computational methods have affirmed the inhibitory activity of the sulfonanilide derivatives and therefore their chemical features were employed to retrieve the compounds from the databases so that identified lead candidates might be imbibed with the same or enhanced biological activity. Therefore, these compounds have been utilized to construct the pharmacophore model so that their structural features can be exploited in redeeming the compounds from the database. To secure the predictive ability of the pharmacophore, it was subsequently validated by ROC curve and the decoy set method. The validated pharmacophore was employed to screen the Chembridge database and was then filtered by drug-like property filters. The resultant candidate molecules were subjected to docking. Owing to the molecular interactions with the key residues present at the active site, two Hits were chosen from the database screening. To further delineate the binding mode analysis, reference, compound 22, and the two Hits proceeded to MD simulations conducted for 50 ns. These results suggest that the binding modes of compound 22 and the Hit compounds were in alignment with the reference compound. The RMSD plots of the backbone of the reference and Hits were found to be below 0.25 nm throughout the simulations referring to its stability and the same result was depicted by the radius of gyration and the potential energy profiles. Moreover, the binding free energy calculations also suggest the superiority of the Hits demonstrated by the lowest average binding energies as compared to the reference compound. van der Waals energy, electrostatic energy, and the SASA energy have contributed negatively to the binding energy, while the polar solvation energy has contributed positively to the binding energy. The average binding energy of the reference was observed to be −56.88 kJ/mol, while that of the Hits was computed to be −77.20 kJ/mol, −77.77 kJ/mol, and −84.58 kJ/mol, respectively, for compound 22, Hit1, and Hit2. Delineating the SAR studies rendered informative interpretations which could aid in gaining further insight into the molecules. Out of the 81 chosen inhibitors that were designed amending the structure, Figure 2, at positions A, B, C, and D, it was noticed that the modification of positions B and C have displayed no greater impact in terms of the dock scores [1]. All the 14 compounds, Table 1, that exhibited higher dock scores than the reference molecule were retrieved from A-position modification. Additionally, it was perceived that five inhibitors resulting due to the modification of A-position containing an alkyl group had their IC_50_ values ranging between 1.2 and 3.96 microM. A total of eight inhibitors generated because of the modification of A-position with aryl group dominated the higher dock score list which exhibited an IC_50_ range from 0.52 to 5.85 microM. Moreover, compound 74 (IC_50_ 16.66 microM) generated as a result of the modification of D-position was noticed in the top dock score list. This finding was also in agreement with the results of Su et al. [1] and further confirms the reliability of the computational results. It can therefore be deduced that the A-positions that was modified with alkyl/aryl group plays a crucial role in imparting high dock scores and further the inhibition of aromatase. Compound 22 that displayed the most valuable result was formed because of the insertion of the aryl group. Furthermore, we evaluated the role of the sulfonamide moiety and its interaction with active site. This moiety has played a crucial role in interacting with the key residues of the active site. The residues Thr310 and Ala306 were observed to form the bonds with the O group of the sulfamide moiety. HA atom of Ala306 has joined with the O13 atom of the ligand with carbon hydrogen bond with a bond distance of 2.6 Å. Thr310 on the other hand has rendered a strong hydrogen bond as described earlier, Figure 18.

**Figure 19:**
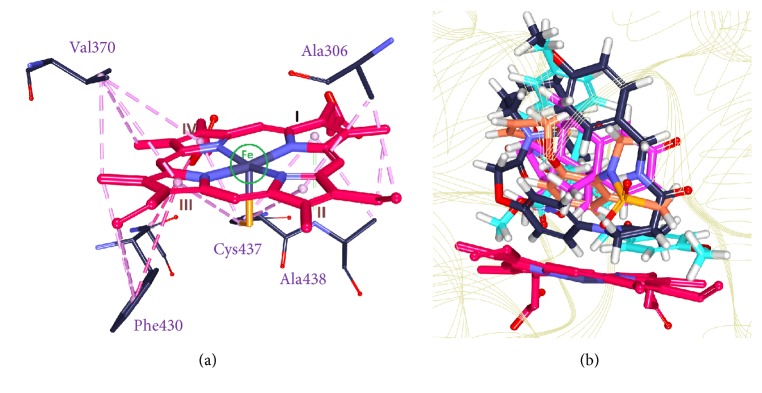
Representation of the protein and the heme group. (a) depicts the interaction of the four pyrrole rings with the residues Ala306, Ala438, Phe430, and Val370, respectively. The four pyrrole rings are labeled in roman numbers; (b) demonstrates the location of the four ligands. The ligands were found to occupy the active site of the protein.

**Figure 20:**
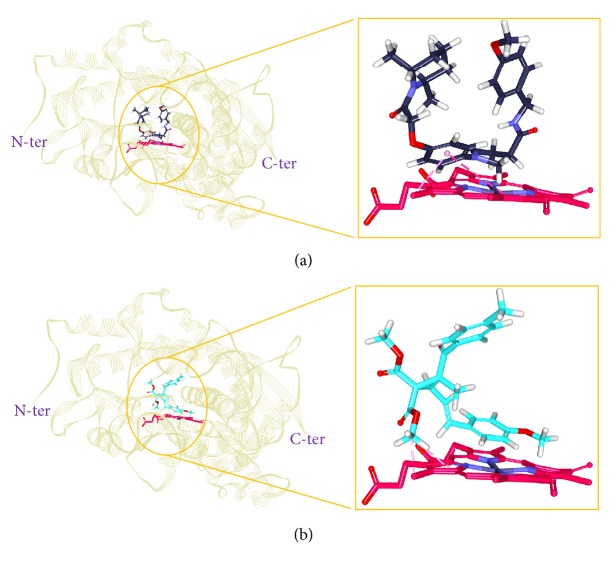
Interaction of the ligands with the heme group. (a) represents the interaction of Hit1 with the heme by *π*-*π* stacked and *π*-alkyl interactions with pyrrole ring III. (b) demonstrates the interaction of Hit2 with pyrrole ring IV by *π*-alkyl and alkyl interactions.

Additionally, we evaluated the interactions of heme with the protein and the Hits. It was observed that the heme was held firmly with Ala306 and Ala438 on one side and Val370 and Phe430 on the other side holding the four pyrrole rings and the Fe group has interacted with the Cys437, Figure 19(a). The HB1 atom Ala306 has interacted with the first pyrrole ring of the heme by the *π*-alkyl hydrophobic interaction with a distance of 4.2 Å. The CB atom of Ala306 has interacted with the CBC atom of the heme forming an alkyl hydrophobic interaction with a distance of 3.5 Å. The second pyrrole ring was held by the HB2 atom of Ala438 forming the hydrophobic *π*-alkyl bond with 4.3 Å. The third pyrrole ring was held by the benzene ring of Phe430 forming *π*-*π* T shaped interaction with a bond distance of 4.9 Å. The fourth pyrrole ring was found to interact with Val370. The CB group of Val370 joins with the heme forming *π*-alkyl hydrophobic interaction with a distance of 5.0 Å, Figure 19(a). Additionally, the residues Trp141, Arg145, and Arg375 were noticed to hold the heme moiety as was represented in the crystal structure [20]. The Hits along with the reference have been found to occupy the active site cavity as was seen in the crystal structure, Figure 19(b). Further we investigated the interactions between the Hits and the heme moiety. Hit1 has formed two bonds with the heme moiety. The benzene ring of Hit1 has interacted with pyrrole ring III of the heme forming *π*-*π* stacked interaction with a bond distance of 5.1 Å. This benzene ring additionally has formed a bond with the CMA group of the heme demonstrated by *π*-alkyl bond with 3.7 Å, Figure 20(a). Hit2 has interacted with the pyrrole ring IV with C30 atom with *π*-alkyl hydrophobic interaction displaying a distance of 3.1 Å. Additionally the C30 atom of Hit2 has rendered the alkyl hydrophobic interaction with a distance of 2.3 Å, Figure 20(b). Focusing on Hit1 and Hit2, it can be understood that the exposed oxygen groups have been involved in the hydrogen bond interactions, while the benzene rings in both the cases were present away from the active site residues. Additionally, it was observed that the Met374 and Asp309 were crucial in forming the hydrogen bond interactions and van der Waals interactions, respectively, with all the ligands and Ile133 was noticed to be involved with the alkyl/*π*-alkyl as was seen in the reference compounds. It can therefore be deduced that these three residues are imperative in inducing the inhibitory activity of the target protein. These results therefore show that the sulfonanilide derivatives can be employed to retrieve novel leads from the databases upon the pharmacophore generation.

Furthermore, the disease gene association and gene-gene association revealed that the CYP19 is a major contributor for several cancer diseases specifically associated with women. Additionally, it was worth observing that the CYP19A1 gene demonstrates its role by being associated with the osteoporosis [56]. As the present drug discovery focuses on identifying the leads against breast cancer, there might be a possibility that these drugs may render positive impact on other cancers as well, a concept seen associated with multidrug targets and drug repositioning [57–61].

## 5. Conclusion

The current article aims at understanding the binding affinities of 81 ligands employing the ligand-based pharmacophore approach. Furthermore, these ligands have ably retrieved the new drug candidates from the Chembridge database. Moreover, the putative binding modes of Hits have been examined upon comparison with the reference compound. Consequently, the obtained Hits might possess the essential inhibitory activities as was seen with the reference (exemestane) and compound 22 (assessed against transfected MCF-7 cells). Furthermore, the MD simulations revealed that the Hits were stable throughout the simulations with no aberrations and additionally the gene network interactions highlight the association of the CYP19 gene with several diseases and therefore it can be understood that these Hits can be used against the associated diseases as well. We therefore believe that the identified Hits are of high therapeutic value and can be used against several diseases. Additionally, the 81 ligands can act as fundamental scaffolds in identifying novel lead candidates from different databases. We further affirm that the adapted methodology can be employed in the identification of new drugs for different diseases.

## Supplementary Material

Supplementary 1. 2D Structures of 81 compounds used for approach 1. All the 81 compounds were docked into the active site of the protein along with the known drugs to evaluate the most potential candidate molecules. Supplementary 2. Dock scores of the known drug molecules. Supplementary 3. Alignment of 14 compounds with the reference molecule. The reference compound is indicated in pink. Supplementary 4: Results of the common feature pharmacophore. Supplementary 5. Structures of known inactive compounds considered for validating the pharmacophore model using ROC.

## Figures and Tables

**Table 1:** Dock scores of 14 inhibitors along with exemestane in the order of their CDOCKER interaction energy. Exemestane showed higher dock score than the other known drugs.

S number	Compound number	-CDOCKER energy (kcal/mol)	-CDOCKER interaction energy (kcal/mol)
1	22	22.54	55.76
2	30	25.63	55.50
3	28	30.32	55.40
4	8	21.03	55.2
5	42	29.80	54.99
6	74	28.80	54.96
7	16	14.36	54.44
8	27	32.14	53.84
9	34	25.96	53.08
10	9	27.97	51.79
11	37	19.56	50.78
12	7	17.91	50.17
13	12	26.78	49.83
14	29	23.32	49.45
	Exemestane	10.25	48.27

**Table 2:** Details of the decoy set method of validation. The GH values obtained conclude that the pharmacophore model is of a good quality.

Parameters	Values
Total number of molecules in database (*D*)	107
Total number of active compounds in database (*A*)	14
Total number of Hit molecules from the database (Ht)	15
Total number of active molecules in Hit list (Ha)	13
% yield of active compounds (Ha/Ht)	0.8
% ratio of active compounds [(Ha/*A*) × 100]	92
Enrichment factor (EF)	6.0
False negatives (*A* − Ha)	1
False positives (Ht − Ha)	2
Goodness of fit score (GF)	0.76

**Table 3:** Dock scores of the Hits and the reference compound.

Compound	-CDOCKER energy (kcal/mol)	-CDOCKER interaction energy (kcal/mol)
Reference	10.25	48.76
Hit1	27.14	64.64
Hit2	32.77	60.04
Compound 22	22.54	55.76

**Table 4:** Interacting residues of the protein and the compounds.

S number	Name	Hydrogen bonds (<3.0 Å)	van der Waals interactions	Alkyl/*π*-alkyl interactions
1	Reference	Arg115, Met374	Phe134, Ile305, Ala306, Asp309, Thr310, Leu372, Leu477, Ser478	Ile133, Trp224, Trp224, Val370, Val373

2	Compound 22	Thr310, Met 374	Phe134, Phe221, Trp224, Asp309, Val374, Leu372, Ser478	Ile133, Val370

3	Hit1	Arg115, Met374	Ile132, Trp224, Ala306, Ile305, Asp309, Thr310, Val369, Val370, Val373, Ala438, Gly439	Ile133, Phe134, Gly436, Cys437, Leu477

4	Hit2	Arg115, Met374	Ile132, Phe134, Phe148, Leu152, Trp224, Met303, Ala306, Asp309, Thr310, Val369, Leu372 Phe430, Gly431, Ser478	Ile133, Phe221, Val370, Val373, Leu477

**Table 5:** Disease associations for query gene.

Disease	Entry name	Score	PMIDs
umls: C0014175	Endometriosis	0.24	35
umls: C0032460	Polycystic ovary syndrome	0.228	23
umls: C1970109	Aromatase excess syndrome	0.207	16
umls: C1458155	Mammary neoplasms	0.199	40
umls: C0033578	Prostatic neoplasms	0.133	7
umls: C0029456	Osteoporosis	0.13	11
umls: C0001418	Adenocarcinoma	0.125	4
umls: C0021361	Female infertility	0.125	3
umls: C0029928	Ovarian diseases	0.123	13

**Table 6:** Genes that share diseases with the query gene.

Gene	Gene name	Diseases
ESR1	Estrogen receptor 1	135
TNF	Tumor necrosis factor	124
IL6	Interleukin 6	123
TP53	Tumor protein p53	115
VEGFA	Vascular endothelial growth factor A	115
AR	Androgen receptor	113
TGFB1	Transforming growth factor, beta 1	113
IGF1	Insulin-like growth factor 1 (somatomedin C)	111
MTHFR	Methylenetetrahydrofolate reductase (NAD(P)H)	108
PTGS2	Prostaglandin-endoperoxide synthase 2 (prostaglandin G/H synthase and cyclooxygenase)	102
