# Supplementary material for: Sulfonanilide Derivatives in Identifying Novel Aromatase Inhibitors by Applying Docking, Virtual Screening, and MD Simulations Studies
Source: Biomed Res Int. 2017 Oct 17;2017:2105610. doi: 10.1155/2017/2105610 (PMC5664374; doi:10.1155/2017/2105610)
Supplement: Supplementary file 1 — Supplementary 1. 2D Structures of 81 compounds used for approach 1. All the 81 compounds were docked into the active site of the protein along with the known drugs to evaluate the most potential candidate molecules. Supplementary 2. Dock scores of the known drug molecules. Supplementary 3. Alignment of 14 compounds with the reference molecule. The reference compound is indicated in pink. Supplementary 4: Results of the common feature pharmacophore. Supplementary 5. Structures of known inactive compounds considered for validating the pharmacophore model using ROC. [file 2105610.f1.zip › Supplementary material/AI_suppl_4.docx]

| \| \| \| \| Hypo no \| Features \| Rank \| Direct Hit \| Partial Hit \| Max Fit \| \| --- \| --- \| --- \| --- \| --- \| --- \| \| 01 \| RHAA \| 134.98 \| 1111 \| 0000 \| 4 \| \| 02 \| RHAA \| 134.54 \| 1111 \| 0000 \| 4 \| \| 03 \| RHAA \| 134.41 \| 1111 \| 0000 \| 4 \| \| 04 \| RHAA \| 133.72 \| 1111 \| 0000 \| 4 \| \| 05 \| RHAA \| 133.27 \| 1111 \| 0000 \| 4 \| \| 06 \| RHAA \| 132.53 \| 1111 \| 0000 \| 4 \| \| 07 \| HAAA \| 131.81 \| 1111 \| 0000 \| 4 \| \| 08 \| HAAA \| 131.57 \| 1111 \| 0000 \| 4 \| \| 09 \| RHAA \| 131.32 \| 1111 \| 0000 \| 4 \| \| 10 \| HAAA \| 130.90 \| 1111 \| 0000 \| 4 \|   Supplementary 4: Results of the common feature pharmacophore \| \| --- \| --- \| --- \| --- \| --- \| --- \| --- \| --- \| --- \| --- \| --- \| --- \| --- \| --- \| --- \| --- \| --- \| --- \| --- \| --- \| --- \| --- \| --- \| --- \| --- \| --- \| --- \| --- \| --- \| --- \| --- \| --- \| --- \| --- \| --- \| --- \| --- \| --- \| --- \| --- \| --- \| --- \| --- \| --- \| --- \| --- \| --- \| --- \| --- \| --- \| --- \| --- \| --- \| --- \| --- \| --- \| --- \| --- \| --- \| --- \| --- \| --- \| --- \| --- \| --- \| --- \| --- \| \| \| --- \| --- \| --- \| --- \| --- \| --- \| --- \| --- \| --- \| --- \| --- \| --- \| --- \| --- \| --- \| --- \| --- \| --- \| --- \| --- \| --- \| --- \| --- \| --- \| --- \| --- \| --- \| --- \| --- \| --- \| --- \| --- \| --- \| --- \| --- \| --- \| --- \| --- \| --- \| --- \| --- \| --- \| --- \| --- \| --- \| --- \| --- \| --- \| --- \| --- \| --- \| --- \| --- \| --- \| --- \| --- \| --- \| --- \| --- \| --- \| --- \| --- \| --- \| --- \| --- \| --- \| --- \| --- \| \| \| --- \| --- \| --- \| --- \| --- \| --- \| --- \| --- \| --- \| --- \| --- \| --- \| --- \| --- \| --- \| --- \| --- \| --- \| --- \| --- \| --- \| --- \| --- \| --- \| --- \| --- \| --- \| --- \| --- \| --- \| --- \| --- \| --- \| --- \| --- \| --- \| --- \| --- \| --- \| --- \| --- \| --- \| --- \| --- \| --- \| --- \| --- \| --- \| --- \| --- \| --- \| --- \| --- \| --- \| --- \| --- \| --- \| --- \| --- \| --- \| --- \| --- \| --- \| --- \| --- \| --- \| --- \| --- \| --- \| |
| --- | --- | --- | --- | --- | --- | --- | --- | --- | --- | --- | --- | --- | --- | --- | --- | --- | --- | --- | --- | --- | --- | --- | --- | --- | --- | --- | --- | --- | --- | --- | --- | --- | --- | --- | --- | --- | --- | --- | --- | --- | --- | --- | --- | --- | --- | --- | --- | --- | --- | --- | --- | --- | --- | --- | --- | --- | --- | --- | --- | --- | --- | --- | --- | --- | --- | --- | --- | --- | --- |
| \|  \| \| --- \| |

RHAA: one aromatic ring, one hydrophobic and two hydrogen bond acceptors

HAAA: hydrophobic and three hydrogen bond acceptors
